# Advancing entropy analysis for heart rate variability: clinical insights for aging and diabetes

**DOI:** 10.3389/fphys.2026.1795118

**Published:** 2026-04-10

**Authors:** Shanglin Yang, Hsientsai Wu, Xuwei Liao, Yuyang Lin, Juin J. Liou

**Affiliations:** 1 School of Electrical and Information Engineering, North Minzu University, Yinchuan, Ningxia, China; 2 Microelectronics and Solid-State Electronics Device Research Centre, North Minzu University, Yinchuan, Ningxia, China

**Keywords:** autonomic function, electrocardiograph (ECG), entropy analysis, heart rate variability (HRV), type 2 diabetes mellitus

## Abstract

Heart rate variability (HRV) entropy analysis is an emerging tool for assessing autonomic function, particularly in older and diabetic populations. Traditional HRV metrics, limited in capturing signal complexity, have been supplemented by advanced entropy methods such as multi-scale entropy (MSE), permutation entropy (PermEn), and the baroreflex entropy index (BEI). In this review, we introduce novel entropy indices, including percussion entropy (PercEn), and explore their potential to enhance HRV assessment. Additionally, we discuss the integration of novel data sources, such as pulse wave velocity (PWV) and crest time, offering an in-depth evaluation of autonomic dysfunction. Performance metrics such as classification accuracy (up to 92.5% in diabetic autonomic dysfunction prediction), sensitivity (87.3%), and specificity (89.1%) demonstrate the potential clinical utility of entropy-based HRV analysis. The key challenges include the prognostic value of entropy metrics, the impact of confounding factors, and the need for standardised methodologies. Advances in machine learning and wearable technology are also examined for real-time HRV monitoring and personalised healthcare. The findings highlight entropy analysis as a promising avenue for autonomic dysfunction assessment, with future research needed to optimise methodologies and establish clinical validation.

## 1 Introduction

Diabetes mellitus (DM) is a prevalent metabolic disorder, affecting approximately 537 million adults globally in 2021, with a global age-standardised prevalence of 6.1% (95% confidence interval, 5.8% to 6.5%) (DF). This chronic condition is characterised by persistent hyperglycaemia resulting from disruption in insulin secretion, insulin action, or both. Individuals with type 2 diabetes mellitus (T2DM), the most common form, are at a heightened risk of complications, including nerve damage (Whitmer et al., 2009; Feldman et al., 2019; American Diabetes Association, 2023) and atherosclerosis (Schmidt et al., 2005; Tsao et al., 2023), and various types of cancer (American Diabetes Association, 2023; Siegel et al., 2023). These complications significantly contribute to the overall morbidity and mortality associated with the disease. A prompt diagnosis and an effective management of diabetes are crucial to mitigate these risks. Among the various complications, autonomic nervous system dysfunction has garnered significant attention due to its profound impact on cardiovascular health. Numerous studies have established a robust link between autonomic dysfunction and an increased risk of cardiovascular diseases (Gerritsen et al., 2001; Sharif et al., 2018; Tsao et al., 2023) and mortality (Benjamin et al., 2019). Autonomic dysfunction in diabetes often manifests as cardiovascular autonomic neuropathy, which can lead to serious consequences such as silent myocardial infarction, cardiac arrhythmias, and sudden cardiac death. Furthermore, reduced autonomic function has predictive value for the accelerated progression of atherosclerosis (Schmidt et al., 2005; Tsao et al., 2023), further complicating the clinical picture for individuals with diabetes. Understanding these associations improves the ability to manage cardiac health in patients with diabetes and may lead to the development of new diagnostic and treatment methods.

Evaluating autonomic function is a critical component in the comprehensive management of patients with diabetes. In clinical settings, autonomic function in older individuals and those with diabetes is typically assessed using a combination of non-invasive tests (Spallone, 2019). These tests focus on various aspects of autonomic nervous system function, particularly heart rate variability (HRV) (Thomas et al., 2019), blood pressure regulation (Mancia and Grassi, 2014), and other physiological responses (Kreibig, 2010). HRV measures the variation in time between successive heartbeats, reflecting autonomic nervous system activity. Continuous electrocardiographic monitoring is used to assess HRV, and various analytical methods, including time-domain, frequency-domain, and non-linear methods (such as entropy analysis), are applied to the electrocardiographic data to evaluate autonomic function. In addition to HRV, blood pressure variability and baroreflex sensitivity are assessed to evaluate the autonomic regulation of blood pressure. Continuous blood pressure monitoring over 24 h allows observation of fluctuations and responses to daily activities. The Valsalva manoeuvre (Ricci et al., 2018), where the patient blows into a mouthpiece to increase thoracic pressure, is used for assessing heart rate and blood pressure responses. The head-up tilt test (Aponte-Becerra and Novak, 2021) involves tilting the patient at different angles while monitoring blood pressure and heart rate to assess autonomic control. In a hospital setting, a detailed history of symptoms such as dizziness, fainting, or palpitations, along with a physical examination, is crucial for clinical evaluation (Swartz, 2014). Standardised questionnaires are used to assess symptoms related to autonomic dysfunction. Additionally, laboratory tests indirectly assess autonomic function by evaluating blood glucose levels, HbA1c, and other markers related to diabetes control and cardiovascular health (Vinik et al., 2013). By combining these methods, clinicians can obtain a comprehensive assessment of autonomic function in older individuals and those with diabetes, enabling improved diagnosis and management of autonomic dysfunction (Spallone et al., 2011).

Non-linear analysis not only captures the complex and chaotic characteristics of HRV but also holds significant potential for autonomic function assessment (Faes et al., 2019). These methods are valuable for revealing intricate patterns and irregularities that time-domain and frequency-domain analyses might overlook (Wang et al., 2018). By analysing the non-linear dynamics of heart rate signals, researchers gain deeper insights into the underlying mechanisms of autonomic regulation. Common non-linear analysis methods include Poincaré plot analysis (Satti et al., 2019; Navarro-Lomas et al., 2020), entropy analysis (Ma et al., 2017; Wu, 2024), and detrended fluctuation analysis (DFA) (Chiang et al., 2016). Poincaré plot analysis provides a visual representation of the correlation between consecutive heartbeats, highlighting the variability and complexity of HRV. Entropy analysis measures the unpredictability and randomness of the heart rate signal, offering a quantitative assessment of the system’s complexity. DFA evaluates the long-term correlations and self-similarity within the HRV signal, revealing fractal-like properties indicative of autonomic control. These non-linear methods allow for a more comprehensive and nuanced understanding of autonomic function, making them indispensable tools in the assessment of cardiovascular health in diabetic and older adult populations. In this context, the use of electrocardiography (ECG) and its entropy analysis has emerged as a valuable tool for assessing autonomic function. Entropy, a measure of complexity and variability, can provide insights into the autonomic regulation of the heart, reflecting the intricate balance between sympathetic and parasympathetic activities. Entropy analysis is a sophisticated tool for understanding the complexity and irregularity of biological signals.

This review aims to systematically examine the applications of entropy-based HRV analysis for assessing autonomic function in older adults and diabetic populations, as reported in published journal articles. By integrating current research findings, this study seeks to advance our understanding of the relation between autonomic dysfunction and cardiovascular risk in the at-risk populations. The insights derived from this review could inform clinical practices and foster further research, potentially leading to improved health outcomes for individuals with diabetes and age-related autonomic decline. Although our findings highlight the promising role of entropy analysis in clinical settings, future research should explore innovative methods to improve diabetes care quality, including the development of wearable ECG devices. Ultimately, a deeper understanding of home-based autonomic function assessment could offer improved diagnostic and therapeutic strategies for identifying individuals at high risk for T2DM.

## 2 Materials and methods

### 2.1 Search strategy and selection criteria

#### 2.1.1 Search strategy and keywords

**Figure 1:**
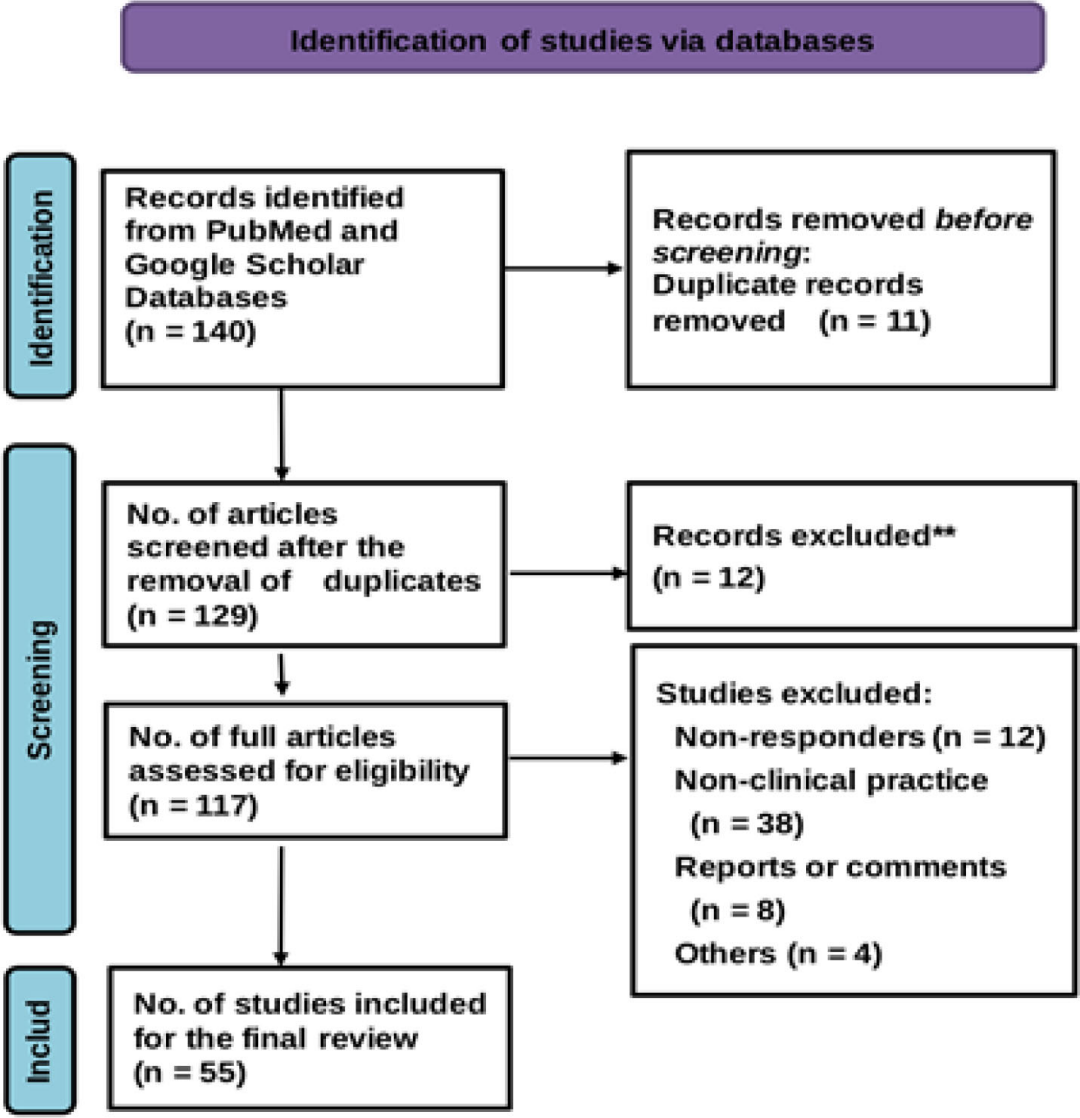
Flow chart showing results of screening for this systematic reviews in accordance with the preferred reporting items for systematic reviews and meta-analyses (PRISMA) guidelines (Wu, 2024).

For this systematic review, we employed Google Scholar and PubMed as the primary databases due to their extensive coverage of scholarly literature across various disciplines, including articles, books, and theses. Both platforms are recognised for their frequent updates, ensuring access to the most recent research. While alternative databases such as Scopus and Web of Science were considered, Google Scholar and PubMed were ultimately chosen for their broad and comprehensive coverage of our specific topic. The search covered all relevant publications from 01 January 2011 to 31 December 2025, using the following pre-determined keywords: (“entropy index” AND “Heart Rate Variability” OR “autonomic function” OR “type 2 diabetes mellitus” in subject terms). A total of 517 records were retrieved from PubMed and 212 from Google Scholar, with 140 duplicate entries identified between the two (**Figure 1**).

#### 2.1.2 Selection criteria

The search was restricted to peer-reviewed journal articles, excluding grey literature and conference proceedings. While non-peer-reviewed sources may provide valuable insights, we prioritised peer-reviewed publications due to their rigorous evaluation process, which ensures a higher standard of quality and reliability. Though this exclusion introduces certain limitations, it allows for a more consistent level of evidence throughout the review. To ensure a thorough and transparent selection process, we followed three key steps: (1) defining explicit inclusion criteria, (2) adhering to the Preferred Reporting Items for Systematic Reviews and Meta-Analyses (PRISMA) guidelines, and (3) prioritising studies with a significant impact. A pre-defined set of criteria, outlined in **Table 1**, was used to assess the relevance of each study. The selection process followed the PRISMA flowchart, shown in **Figure 1**, ensuring full transparency and adherence to systematic review best practices. Additionally, we included only studies with an average annual citation count of at least five, ensuring that our review focused on research with demonstrable influence in the field. The final pool of studies was selected based on their originality, relevance, and contribution to the broader context of the review. A total of 55 studies met these criteria and were included in the final analysis (**Figure 1**, **Table 1**).

**Table 1:** Inclusion and exclusion criteria.

Inclusion criteria	Exclusion criteria
Entropy analysis relative Published in English since 2012;	Entropy analysis relative Published in English before 2011
Investigated HRV in the aged and diabetic for entropy analysis data	Non-responders: Investigated the different diseases
Included all entropy analysis HRV relative studies: observational, cross-sectional studies, and controlled trials.	Non-Clinical Practice: Case report or meta-analysis
Published journal by peer review	Comments or Case report: Conference paper, grey literature and unpublished studies
Studies written in English	Non-English: Studies written in other language

### 2.2 Development trend of entropy methods for HRV assessment

The assessment of heart rate variability (HRV) using entropy methods has evolved significantly, driven by the need for more precise and comprehensive tools to evaluate autonomic function. These advancements can be broadly categorised into innovations in entropy indices, the integration of novel data sources, advancements in computational techniques, and the exploration of clinical applications, particularly in aging and diabetic populations.

#### 2.2.1 Novelty in indices

Traditional entropy measures such as approximate entropy (ApEn) and sample entropy (SampEn) have been pivotal in HRV analysis (Richman and Moorman, 2000). However, their limitations, including sensitivity to data length and noise, have led to the development of new indices. Multi-scale entropy (MSE) (Costa et al., 2002; Costa et al., 2005; Ho et al., 2011), fuzzy entropy (FuzzyEn) (Liu et al., 2013), permutation entropy (PermEn) (Bian et al., 2012), and percussion entropy (PercEn) (Wei et al., 2019b) are among the emerging indices gaining attention.

MSE represents a significant advancement in HRV analysis by evaluating entropy across multiple time scales, capturing both short-term and long-term patterns in HRV data. This approach provides a more comprehensive assessment of physiological complexity, enabling the detection of subtle autonomic changes that may be overlooked by single-scale entropy methods (Karmakar et al., 2020). FuzzyEn improves the stability of entropy estimates through a fuzzy membership function, which smooths the transition between data points (Xiang et al., 2015). This method addresses the abrupt categorisation issues inherent in ApEn and SampEn, making it particularly useful in clinical settings with limited or noisy data, enhancing the reliability of HRV analysis. PermEn, designed to quantify the complexity of time series data, stands out for its robustness against noise and non-stationarity. It is computed based on the ordinal patterns of the data rather than their amplitude, making it a powerful tool in scenarios where signal quality is compromised (Song et al., 2018). PermEn has shown promise in differentiating various physiological states, including varying levels of autonomic dysfunction in diabetic and older adult populations (Bian et al., 2012). PercEn, a recent addition to the field (Yang et al., 2024), focuses on capturing sudden changes or ‘percussions’ in heart rate signals. Unlike other entropy methods, PercEn detects abrupt signal changes, assessing complexity by identifying transient variations. This method is particularly valuable for detecting short-term fluctuations and anomalies in autonomic function, which are crucial in complex pathological conditions often seen in aging and diabetic populations (Xing et al., 2023).

These novel indices enhance sensitivity and specificity in detecting autonomic dysfunction and facilitate the differentiation between various pathological conditions. This differentiation is crucial for clinical diagnosis and monitoring, especially for older adults and those with diabetes, who may present with overlapping symptoms and risk factors. **Table 2** summarises various methodologies related to entropy analysis for assessing HRV, particularly focusing on aged and diabetic populations, highlighting the advantages, limitations, and specific uses.

**Table 2:** Summary of entropy-based HRV studies, highlighting their advantages, limitations, and uses.

Methodology/technique	Advantages	Limitations	Uses
ApEn (Richman and Moorman, 2000)	Non-invasive, effective for HRV analysis in diabetes	Sensitive to parameter selection	Assessing cardiovascular autonomic function in patients with diabetes
SampEn (Richman and Moorman, 2000)	Provides insights into RR interval dynamics	May not capture long-range correlations	Evaluating autonomic dysfunction in T2DM
PermEn (Bian et al., 2012; Song et al., 2018)	Assesses HRV complexity from an ordinal perspective	Requires specific temporal scales	Diagnosing cardiovascular autonomic neuropathy
MSE (Costa et al., 2002; Costa et al., 2005; Ho et al., 2011)	Captures complexity across multiple time scales	Computationally intensive	Analysing HRV changes in cardiovascular diseases
FuzzyEn (Liu et al., 2013; Xiang et al., 2015)	Handles uncertainty in data well	Complexity in implementation	Monitoring HRV in aged and diabetic populations
PercEn (Yang et al., 2024)	Quantifies irregularity in HRV	Interpretation can be complex	General assessment of heart health in aging and diabetic populations

ApEn, approximate entropy; FuzzyEn, fuzzy entropy; HRV, heart rate variability; MSE, multiscale entropy; PercEn, percussion entropy; PermEn, permutation entropy; SampEn, sample entropy; T2DM, type 2 diabetes mellitus.

#### 2.2.2 Novelty in HRV parameterization

Recent developments in HRV parameterization have expanded the range of physiological signals used to derive HRV-related metrics, moving beyond the traditional reliance on electrocardiography (ECG)-based R–R intervals. Photoplethysmographic signals obtained from wearable devices have emerged as an important alternative source for HRV assessment (Stone et al., 2021). Photoplethysmography (PPG) measures blood volume changes in the microvascular bed of tissue and offers a less invasive and more accessible approach than electrocardiography, particularly for continuous and long-term monitoring (Elgendi et al., 2019).

Although the accuracy of photoplethysmography (PPG) for HRV analysis was historically constrained by motion artefacts and lower signal fidelity compared with ECG, recent advances in signal processing algorithms—such as adaptive filtering and machine learning-based noise reduction—have improved the reliability of PPG-derived HRV measurements (Jong et al., 2017). These improvements have broadened the applications of PPG-based HRV analysis from controlled clinical environments to consumer health devices and wearable monitoring platforms. Pulse wave velocity (PWV), calculated from synchronised digital volume pulses of the toe and ECG, is gaining attention as an important data source in HRV analysis (Nakamura et al., 2010). PWV measures the speed of pressure waves traveling through the arterial system, which is indicative of arterial stiffness. Increased arterial stiffness is a marker of cardiovascular risk and can be associated with autonomic dysfunction. Incorporating PWV into HRV analysis offers additional insights into cardiovascular and autonomic systems, as PWV reflects the integrative effects of arterial health on heart rate dynamics (Wu et al., 2011). Crest time, defined as the time interval from the foot point of the wave to the first peak, provides information about the shape and duration of the arterial pulse wave (Wu et al., 2010). Changes in crest time can indicate alterations in arterial compliance and reflect underlying changes in autonomic regulation. Integrating crest time with HRV analysis enhances understanding of the interaction between arterial function and HRV, offering a more comprehensive assessment of cardiovascular health (Gupta et al., 2022). Continuous blood pressure monitoring is another innovative data source. When combined with HRV analysis, it provides additional insights into autonomic function by revealing beat-to-beat variations that correlate with HRV, offering a more comprehensive view of cardiovascular and autonomic dynamics (Tiwari et al., 2021).

Using various types of data, such as electrocardiographic data, photoplethysmographic data, PWV, crest time, and blood pressure signals, offers a more comprehensive assessment for HRV assessment (Jamin and Humeau-Heurtier, 2019). This multimodal approach helps to check and improve the accuracy of entropy measures, making the evaluation of autonomic function reliable. Moreover, advanced techniques such as deep learning and data fusion help extract detailed information from the different data sources, adding depth to the analysis. The increasing incorporation of novel physiological signal sources and derived indices has facilitated the development of expanded HRV parameterization frameworks, supporting more comprehensive and continuous assessment of autonomic function through wearable and non-invasive monitoring technologies (Shaffer and Ginsberg, 2017; Ding et al., 2020; Bayoumy et al., 2021; Li et al., 2023). For aging and diabetic populations, these advancements provide critical tools for early detection and management of autonomic dysfunction, potentially improving clinical outcomes and quality of life (Bönhof et al., 2022). In addition to integrating multiple physiological signal sources, recent research has also explored novel parameterisation strategies derived from ECG signals to enhance HRV analysis. One such example is a novel input parameter—the T-R interval (TRI) (Yang et al., 2025)—to allow readers to better understand its comparison with the traditional input parameter, R-R interval [RRI], and their different effects on four HRV assessment indices (low-frequency power [LFP] to high-frequency power [HFP] ratio [LHR], standard deviation 1 [SD1] and standard deviation 2 [SD2] ratio [SSR], MSE, and baroreflex entropy index [BEI]). It presents a novel input parameter, the T wave to R wave interval (TRI) [as illustrated in (Equations 1, 2)], this parameterisation approach extends conventional R–R interval–based HRV analysis by incorporating additional morphological information from the ECG waveform, thereby providing further insights into autonomic dysfunction in aging and diabetic populations.

(1)  TRI=RRI–RTc

(2) Where RTc={rt1, rt2,…,rti+1,…,rtiN}, while rti+1=RTi+1/RRIi+1, RRI's unit is seconds.

#### 2.2.3 Advancements in computational techniques

The application of advanced computational techniques has significantly enhanced the capabilities of entropy methods in HRV assessment (Yang et al., 2025). The key developments include the use of machine learning algorithms, both supervised and unsupervised, for feature selection, classification, and prediction tasks, allowing for more precise differentiation of autonomic dysfunction across various populations.

Deep learning techniques (Al-Qazzaz et al., 2024), particularly convolutional neural networks (CNNs) (Gibert et al., 2019) and recurrent neural networks (RNNs) (Martínez-García et al., 2020), have been employed to automatically extract features from HRV data. These methods can capture complex temporal patterns and interactions in the data, which are often missed by traditional statistical methods. Integrating these deep learning models with entropy measures has led to improved diagnostic accuracy and efficient handling of large datasets. Data fusion techniques, which integrate multiple physiological signals such as electrocardiographic, photoplethysmographic, and blood pressure, provide a comprehensive analysis of autonomic function. By combining data from different modalities, these techniques enhance the robustness and accuracy of HRV assessments, offering a more holistic view of the cardiovascular and autonomic systems (Pham et al., 2021).

The development of real-time signal processing and analysis platforms has enabled continuous HRV monitoring in various settings, from clinical environments to wearable devices. These platforms leverage computational advancements to provide immediate feedback on autonomic function, which is particularly valuable for managing chronic conditions such as diabetes and hypertension.

#### 2.2.4 Exploration of clinical applications in aging and diabetic populations

The advancements in entropy methods and computational techniques have paved the way for exploring clinical applications, particularly in aging and diabetic populations. These groups are often at high risk of autonomic dysfunction, making early detection and monitoring critical (Cossul et al., 2023).

In aging populations, HRV analysis using advanced entropy methods has shown potential for identifying early alterations in autonomic regulation that may precede clinical manifestations of cardiovascular diseases (Yang et al., 2025), neurodegenerative disorders (Averna et al., 2023), or other age-related conditions (Yang et al., 2020). However, most current evidence derives from observational and exploratory studies, and further prospective clinical validation is required before these entropy-based HRV measures can be widely adopted for early diagnostic applications. By assessing the complexity of HRV, clinicians can gain insights into the overall health of the autonomic nervous system and implement preventive or therapeutic interventions accordingly (Wei et al., 2020b). In diabetic populations, assessing autonomic function through HRV entropy analysis is particularly relevant due to the high prevalence of diabetic autonomic neuropathy (DAN) (Li et al., 2023). Early detection of DAN based on subtle changes in HRV patterns can guide effective management strategies, including lifestyle modifications, medication adjustments, and monitoring of glycaemic control. Advanced entropy methods, combined with continuous monitoring technologies, provide a non-invasive and sensitive approach to tracking the progression of autonomic dysfunction in patients with diabetes (Daskalaki et al., 2022).

Moreover, wearable devices equipped with HRV monitoring capabilities offer new possibilities for home-based care and long-term monitoring (Bayoumy et al., 2021). This is especially beneficial for older patients and those with diabetes who require ongoing assessment of their autonomic function. The integration of these devices with telemedicine platforms further enhances patient care by allowing healthcare providers to remotely monitor and respond to changes in autonomic function (Ding et al., 2020).

These clinical applications highlight the potential of entropy-based HRV analysis to improve patient outcomes through early detection, personalised treatment, and continuous monitoring. As research progresses, further refinement of these techniques and their integration into clinical practice will likely enhance their utility and accessibility.

### 2.3 HRV methods (LHR, SSR, MSE, and BEI) for aged and diabetics

•LF/HF ratio (LHR) (Shaffer and Ginsberg, 2017).

LHR is a widely used metric in HRV analysis (Chen et al., 2019), providing valuable insights into the balance between the sympathetic and parasympathetic nervous systems (Lin et al., 2022). LFP and HFP are typically derived from the power spectral density of RRI time series.

•Poincaré plot index.

Another important method in HRV analysis is the Poincaré plot (Haryadi et al., 2018), a graphical representation that elucidates heart rate dynamics by plotting each R-R interval against the subsequent one. This plot yields two key measures—SD1 and SD2. SD1 reflects the dispersion of points perpendicular to the line of identity (where consecutive R-R intervals are equal) and represents short-term HRV, which is often associated with parasympathetic activity. SD2 indicates the dispersion of points along the line of identity, encompassing both short- and long-term HRV and reflecting overall autonomic influence. SSR (SD1/SD2 ratio) serves as an index of the balance between short- and long-term HRV components, with higher SSR values indicating dominant parasympathetic influence and lower values suggesting a greater contribution from sympathetic activity or a balanced autonomic influence (Wu et al., 2013). This metric is particularly useful in assessing autonomic nervous system function and identifying potential abnormalities in HRV analysis.

•Entropy analysis indices.

A. Small-scale MSE indices: non-linear HRV index.

MSE analysis provides a comprehensive approach to HRV assessment by evaluating entropy across different timescales. Small-scale entropy indices primarily reflect short-term heart rate fluctuations in HRV analysis, whereas large-scale entropy indices capture longer-term regulatory dynamics of cardiovascular control systems. Multiscale entropy analysis has been widely applied to ECG-derived HRV signals to evaluate physiological complexity across different temporal scales (Valencia et al., 2009).

B. BEI for PercEn analysis: a non-linear HRV index.

The BEI in (Equations 3, 4) is a non-linear HRV measure designed to assess the complexity and adaptability of the baroreflex mechanism. BEI quantifies the entropy or unpredictability of beat-to-beat changes in the heart rate regulated by baroreceptors, offering insights into autonomic dysfunction. Higher BEI values reflect greater physiological adaptability, whereas lower BEI values suggest impaired baroreflex sensitivity and autonomic dysregulation (Yang et al., 2024). The degree of similarity (DS) in (Equation 3) in the fluctuation vectors between two time series (the RRI series and the Amp series of lead II ECG) for each scale factor is as follows:

(3) $${\text{ DS}}_{\text{s}}^{\text{m}}\;\;=\;\frac{1}{\left(\text{n}-\text{m}-\text{s}+1\right)}\sum_{\text{i}=1}^{\text{n}-\text{m}-\text{s}+1}\text{count}(\text{i}),\;\;\;\;\;$$

where m is the dimension of the embedded vector, and count(i) acts as a similar fluctuation number between the two-time series. BEI is calculated as shown in (Equation 4) (Yang et al., 2024):

(4) $$\begin{array}{c} \text{BEI}=\;ln[\frac{\;\sum_{\text{s}=1}^{3}{\text{DS}}_{\text{s}}^{\text{m}=2}+\sum_{\text{s}=1}^{3}{\text{DS}}_{\text{s}}^{\text{m}=3}+\sum_{\text{s}=1}^{3}{\text{DS}}_{\text{s}}^{\text{m}=4}}{\;\sum_{\text{s}=4}^{5}{\text{DS}}_{\text{s}}^{\text{m}=2}+\sum_{\text{s}=4}^{5}{\text{DS}}_{\text{s}}^{\text{m}=3}+\sum_{\text{s}=4}^{5}{\text{DS}}_{\text{s}}^{\text{m}=4}}],\\ ln\;\text{is}\;\text{natural logarithmic operator}. \end{array}$$

## 3 Impact of entropy analysis in HRV

### 3.1 HRV methods (LHR, SSR, MSE, and BEI)

To illustrate the differences between traditional HRV assessment methods and entropy-based approaches, **Table 3** provides a comprehensive overview of four widely used HRV assessment indices—LHR, SSR, MSE, and BEI—focusing on their respective advantages, limitations, and clinical uses. These indices are essential tools in evaluating autonomic function and cardiovascular health, particularly in aging and diabetic populations. The table highlights the distinct characteristics of each method, offering insights into their strengths in diagnosing specific conditions, as well as their limitations in terms of sensitivity, computational demands, and applicability. By examining these indices in detail, this table aids in understanding their role in enhancing diagnostic accuracy across various medical fields.

**Table 3:** Summary of four HRV assessment indices (LHR, SSR, MSE, and BEI), highlighting their advantages, limitations, and uses.

Index	Advantages	Limitations	Uses
LHR (low-to-high frequency ratio) (Chen et al., 2019; Lin et al., 2022)	- Simple to calculate from power spectral density - Reflects autonomic balance (sympathetic vs. parasympathetic activity)	- Sensitive to breathing rate and environmental factors - Limited in reflecting complex HRV changes	- Assessing autonomic balance - Detecting stress and fatigue
SSR (symmetry of successive RR intervals) (Wu et al., 2013; Haryadi et al., 2018)	- Captures fine asymmetry in RR intervals - Sensitive to subtle cardiac variability	- Requires clean RR interval data - Less widely studied than other indices	- Early detection of arrhythmias - Evaluating heart rate variability (HRV) in post-cardiac event recovery
MSE (multi-scale entropy) (Valencia et al., 2009)	- Analyses HRV across multiple time scales - Robust for complex and non-linear data	- Computationally intensive - Requires careful selection of parameters for coarse-graining	- Exploring long-term HRV trends - Studying physiological responses in aging and disease
BEI (Baroreflex entropy index) (Yang et al., 2024)	- Specifically evaluates autonomic-baroreflex interactions - Applicable in cardiovascular studies	- Limited to populations with baroreflex-related issues - Requires specialised data preprocessing	- Assessing baroreflex sensitivity in patients with diabetes - Monitoring cardiac autonomic dysfunction.

### 3.2 Long-term prognostic value of ECG entropy analysis

The long-term prognostic value of ECG entropy analysis remains a significant area of research, particularly for its potential in predicting cardiovascular outcomes in various populations. The following section explores the findings from several key studies that have investigated the role of ECG entropy in prognostication, highlighting both the promise and the challenges associated with this approach.

One of the pioneering studies in this field, conducted by Ho et al. (2011) (Xing et al., 2023), aimed to investigate the prognostic value of parameters derived from MSE in patients with systolic heart failure. The study found that MSE could serve as a potential prognostic marker, offering insights into the autonomic regulation of HRV and its implications for patient outcomes. However, the study also underscored the need for standardisation in ECG entropy analysis methods, as variations in entropy calculation, data processing techniques, and analysis parameters can lead to issues with comparability and reproducibility of results. Chen et al. (2021) (Chen et al., 2021) explored the application of wrist waveform analysis during post-occlusive reactive hyperaemia for assessing the risk of atherosclerosis. The study utilised digital volume pulse and HRV information to derive indices such as the diastolic index and pulse entropy index (PEI), which were used to stratify atherosclerotic risk in young, healthy individuals. Their findings indicated that waist circumference and the PEI of the wrist pulse wave during post-occlusive reactive hyperaemia are significant factors for early identification of individuals at high risk of cardiovascular disease. This suggests that ECG entropy metrics could play a crucial role in early risk stratification for atherosclerosis, providing a non-invasive and cost-effective screening tool.

The use of HRV signals for automated classification of diabetes was reviewed by Adam et al. (2017) (Adam et al., 2017), who discussed various techniques for feature extraction and the efficiency of different classification systems. The review highlighted that the choice of entropy metrics and the methods used for their computation can significantly influence the classification outcomes, underscoring the importance of methodological consistency in studies utilising ECG entropy analysis for prognostication.

Haryadi et al. (2018) (Haryadi et al., 2018) demonstrated the enhanced sensitivity of the multi-scale Poincaré plot in distinguishing between individuals with and without diabetes, compared to traditional measures like short-term scaling exponent (SSR) and MSE. This study emphasised the utility of advanced entropy-based techniques in improving the differentiation of diabetic conditions, which is critical for the timely intervention and management of diabetes-related complications. Mishra and Nirala (2023) (Mishra and Nirala, 2023) proposed a non-invasive T2DM classification system using short photoplethysmographic signals and various machine learning techniques. The study’s findings supported the feasibility of using short-duration signals to accurately classify diabetic status, thereby offering a practical approach for large-scale screening and monitoring.

Further research by Wei and colleagues explored the use of PercEn analysis and digital volume pulse measured at the fingertip as prognostic indicators for future peripheral neuropathy in patients with T2DM. These studies (Wei et al., 2019; Wei et al., 2020) (Wei et al., 2019a; Wei et al., 2020b; Wei et al., 2020a) demonstrated that entropy analysis of synchronised ECG and PPG signals could serve as a valuable tool in predicting diabetic peripheral neuropathy, providing early warnings and facilitating preventive care strategies.

Collectively, these studies highlight the potential of ECG entropy analysis as a prognostic tool for various health conditions, including heart failure, diabetes, and atherosclerosis. However, the standardisation of analysis methods remains a critical challenge. Variations in entropy calculation, data processing, and analysis parameters can affect the comparability and reproducibility of results, underscoring the need for consensus in methodological approaches. Future research should focus on refining these methods to enhance their prognostic accuracy and reliability, ensuring they can be effectively integrated into clinical practice.

### 3.3 Impact of multiple confounding factors on ECG entropy metrics

The interpretation of entropy metrics derived from ECG signals can be significantly influenced by multiple confounding factors, which necessitate careful consideration in clinical and research settings. This section explores the effects of various confounders on ECG entropy measures and highlights the potential implications for patient assessment, particularly in aging and diabetic populations.

One notable study by Ho et al. (2011) (Ho et al., 2011) examined the prognostic value of MSE in patients with congestive heart failure (CHF). The findings indicated that the area under the MSE curve for scales 6 to 20 was not significantly affected by the administration of β-blockers, suggesting that these specific MSE measures may provide independent prognostic value in patients with CHF. This finding highlights the importance of considering the effects of medication when interpreting ECG entropy metrics for risk stratification.

In another investigation, Wu et al. (2013) (Wu et al., 2013) assessed autonomic dysfunction in patients with diabetes using reactive hyperaemia and wrist-acquired waveform signals. The study demonstrated that ECG entropy measures could successfully distinguish between healthy individuals and those with diabetes, illustrating the method’s sensitivity to autonomic changes induced by diabetes. However, the influence of various physiological states, such as blood glucose levels and vascular reactivity, must be considered when analysing entropy metrics in diabetic populations.

Further, Lees et al. (2018) (Lees et al., 2018) provided compelling evidence that HRV parameters, including entropy-based measures, have potential utility as biomarkers for predicting stroke and post-stroke complications. They emphasised the need for integrating non-linear and novel HRV parameters, as well as employing advanced statistical methods, including predictive regression and hazard modelling. This underscores the complexity of interpreting HRV and ECG entropy metrics, as they may be influenced by a myriad of factors including stroke severity, treatment modalities, and patient recovery phases. Therefore, utilising electroencephalographic and electrocardiographic data, small scale MSE may serve as a simple preliminary screening tool for evaluating the severity of OSA before proceeding to PSG analysis (Pan et al., 2015). Lastly, a systematic review by Zanelli et al. (2022) (Zanelli et al., 2022) explored the application of ECG and PPG analysis in diabetes care. The review highlighted the potential of these techniques in detecting and managing diabetes while also pointing out the challenges in achieving clinical validation and standardisation of data processing protocols. The variability in photoplethysmographic and electrocardiographic signal acquisition methods, along with patient-specific factors such as skin tone and tissue perfusion, can significantly affect the accuracy and reliability of entropy measures in clinical practice.

These studies collectively underscore the necessity of accounting for confounding factors when utilising ECG entropy metrics in clinical and research contexts. Factors such as medication use, physiological state, and methodological differences in signal acquisition and processing can all influence the outcomes of entropy analysis, thus impacting the reliability and validity of these measures for patient assessment. Future research should continue to refine these metrics, ensuring they are robust against such confounders, and explore their potential applications in diverse patient populations.

### 3.4 Underlying mechanisms by which changes in entropy reflect autonomic dysfunction

A growing body of evidence supports the hypothesis that changes in entropy metrics are reflective of underlying autonomic dysfunction, particularly in conditions such as T2DM. Inflammation has been consistently linked to decreased blood pressure reflex sensitivity, a key indicator of autonomic dysfunction. For instance, Papaioannou et al. (2008) demonstrated that heart rate and blood pressure variability, alongside baroreflex sensitivity, are significantly affected in patients with acute brain injury, highlighting the role of autonomic regulation in inflammatory states (Papaioannou et al., 2008). In the context of T2DM, heightened inflammatory burden has been recently characterised as a significant factor. Aktas (2023) found a strong association between the prognostic nutritional index and chronic microvascular complications in patients with T2DM, underscoring the role of inflammation in the disease’s progression (Aktas, 2023). Moreover, Basaran and Aktas (2024) reported that inflammatory markers were markedly elevated in patients with poor glycaemic control, further linking inflammation to autonomic dysregulation in T2DM (Basaran and Aktas, 2024).

Investigating how changes in entropy reflect autonomic dysfunction necessitates considering how HRV entropy provides insights into the central autonomic network. Riganello et al. (2018) demonstrated that HRV entropy can discriminate between different disorders of consciousness and correlates with resting-state functional magnetic resonance imaging brain connectivity of the central autonomic network, highlighting its utility in understanding autonomic regulation (Riganello et al., 2018). Deschodt-Arsac et al. (2020) found that entropy in heart rate dynamics can effectively reflect improvements in neurovisceral complexity resulting from HRV-biofeedback training, particularly during stress-cognition interactions (Deschodt-Arsac et al., 2020). This highlights the sensitivity of entropy-based measures to changes in autonomic regulation related to cognitive and stress responses. Additionally, Jelinek et al. (2021) compared various entropy measures to detect the progression of autonomic neuropathy, emphasising the utility of diffusion entropy over MSE and Renyi entropy (Jelinek et al., 2021).

Xiao et al. (2019) corroborated the aforementioned findings by applying a modified entropy method to assess the complexity of baroreflex sensitivity in healthy individuals and those with diabetes, demonstrating significant correlations with inflammatory conditions (Xiao et al., 2019). Given the well-documented inflammatory profile of T2DM, its complications, and associated cardiac diseases, further investigation into BEI within this context is warranted. For asymptomatic individuals, a lower BEI value may serve as a crucial biomarker associated with an increased risk of developing T2DM, as suggested by logistic regression analyses (**Table 3**). This study identifies BEI as a significant protective factor against T2DM, with potential implications for predicting cardiovascular complications and guiding therapeutic interventions (Yang et al., 2024).

In summary, the interplay between inflammation and autonomic dysfunction, as reflected by changes in entropy metrics such as BEI, underscores the importance of integrating these measures into clinical practice. Future research should focus on the longitudinal monitoring of BEI to evaluate its effectiveness in managing autonomic dysfunction and improving long-term health outcomes in patients with T2DM.

### 3.5 Training and expertise required for healthcare professionals to accurately interpret and utilise entropy analysis

The integration of entropy analysis in clinical settings necessitates specialised training and expertise among healthcare professionals. The complexity of entropy-based techniques, particularly in HRV assessment, requires a thorough understanding of the underlying mathematical principles and their practical applications in patient care. A comprehensive review by Seoni et al. (2023) (Seoni et al., 2023) underscores the importance of incorporating uncertainty quantification in healthcare, particularly in machine learning and deep learning models. The study emphasised that such techniques can enhance the accuracy and reliability of medical diagnoses by providing valuable insights into the management of uncertainty in real-world medical data. This approach necessitates that healthcare professionals not only understand entropy-based methods but also appreciate their role in augmenting decision-making processes, as highlighted by Kang et al. (2021) (Kang et al., 2021). Their work introduces an uncertainty-based clinician-in-the-loop framework, which has been shown to improve the accuracy and confidence of medical classifications in a cost-effective manner.

Furthermore, Imran et al. (2022) (Imran et al., 2022) focused on the application of entropy enhancement techniques in medical image analysis, specifically for detecting diabetic retinopathy. They addressed challenges, including subtle feature detection and imbalance in datasets, proposing a novel hybrid neural network approach. This underscores the need for clinicians to be adept in interpreting complex data outputs generated from advanced computational techniques. Cheng et al. (2023) (Cheng et al., 2023) demonstrated the significance of HRV metrics during different sleep stages in assessing autonomic nervous system dysfunction and metabolic function in patients with T2DM. Their findings suggest that healthcare professionals need to be trained to understand and interpret HRV data in the context of sleep physiology, as these metrics are particularly relevant for glycaemic control and overall metabolic assessment.

Wei et al. (2020) and Xiao et al. (2021) (Wei et al., 2020b; Xiao et al., 2021) delve into the application of PercEn in predicting future peripheral neuropathy (DPN) in patients with T2DM. Additionally, a recent study by Wu et al. (2023) (Wu et al., 2023) demonstrated that the skin carotenoid score, derived from daily vegetable and fruit consumption, along with the HRV_index with PercEn, are significant predictors for the onset of T2DM in middle-aged individuals, eliminating the need for blood samples. Their studies highlight that smaller PE values are significant predictors of the risk of T2DM and/or DPN, thereby offering a prognostic tool that requires precise interpretation by trained professionals. The adoption of such novel entropy-based approaches in clinical practice underscores the need for a robust educational framework to equip healthcare providers with the necessary skills. **Table 4** highlights the quantitative improvements in diagnostic accuracy achieved through entropy-based HRV analysis across various medical fields. These advancements are evident in cardiology, endocrinology, neurology, pulmonology, geriatrics, and critical care. The table underscores how specific HRV entropy metrics, such as MSE, ApEn, and BEI, have enhanced diagnostic capabilities. The listed studies showcase the application of these metrics in improving disease detection, risk stratification, and patient monitoring, supported by relevant references that demonstrate the growing significance of HRV entropy methods in clinical practice.

**Table 4:** Quantitative improvements in diagnostic accuracy across various medical fields using entropy-based HRV analysis.

Medical field	Diagnostic improvement	Key HRV entropy metrics	Relevant references
Cardiology	Enhanced risk stratification in patients with myocardial infarction	Multiscale Entropy (MSE), Sample Entropy (SampEn)	(Frassineti et al., 2021) (Liang et al., 2021)
Endocrinology	Improved detection of autonomic dysfunction in type 2 diabetes mellitus	Approximate Entropy (ApEn), Baroreflex Entropy Index	Shanglin et al (Yang et al., 2024) (Yu et al., 2024)
Neurology	More accurate differentiation between Parkinson’s disease and healthy controls	Permutation Entropy, MSE	Mayor et al., 2023) (Jelinek et al., 2021)
Pulmonology	Early detection of sleep apnoea through subtle variability patterns in respiratory HRV	SampEn, Complexity Index	(Chattopadhyay and Das, 2022) (Jeena et al., 2025)
Geriatrics	Enhanced prognostic assessment of frailty and fall risk in aging populations	MSE, Shannon Entropy	Ackun et al (Shokouhmand et al., 2021) (Dorantes-Méndez et al., 2022)
Critical Care	Early prediction of sepsis and organ dysfunction in patients admitted in ICU	ApEn, SampEn	(Bodenes et al., 2022) (Zeiler et al., 2021)

### 3.6 Summary of figures on entropy methods, clinical applications, and physiological signal sources

**Figure 2:**
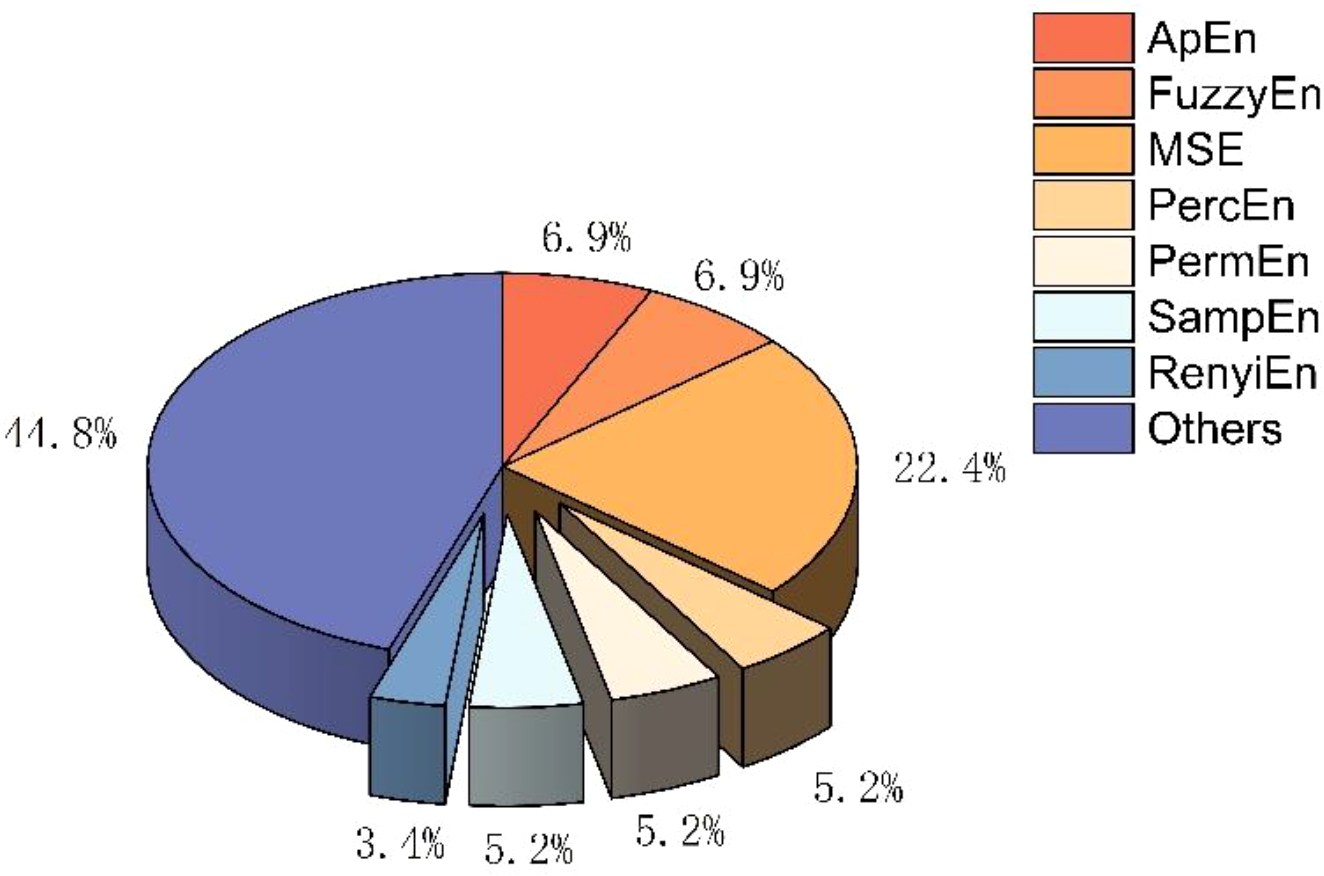
Distribution of entropy methods used in heart rate variability (HRV) studies included in the review.

This 3-dimensional pie chart (**Figure 2**) illustrates the proportional use of major entropy-based analytical methods across the studies reviewed. Approximate entropy (ApEn) and fuzzy entropy (FuzzyEn) each account for 6.9% of reported applications, while multi-scale entropy (MSE) represents 22.4%. Novel indices such as percussion entropy (PercEn) and permutation entropy (PermEn) contribute 5.2% each. Sample entropy (SampEn) represents 5.2%, and Rényi entropy accounts for 3.4%. The remaining 44.8% comprises other entropy-based or hybrid non-linear HRV metrics. This distribution highlights the methodological diversity in contemporary HRV entropy analysis and the increasing adoption of advanced non-linear indices in aging and diabetic populations.

**Figure 3:**
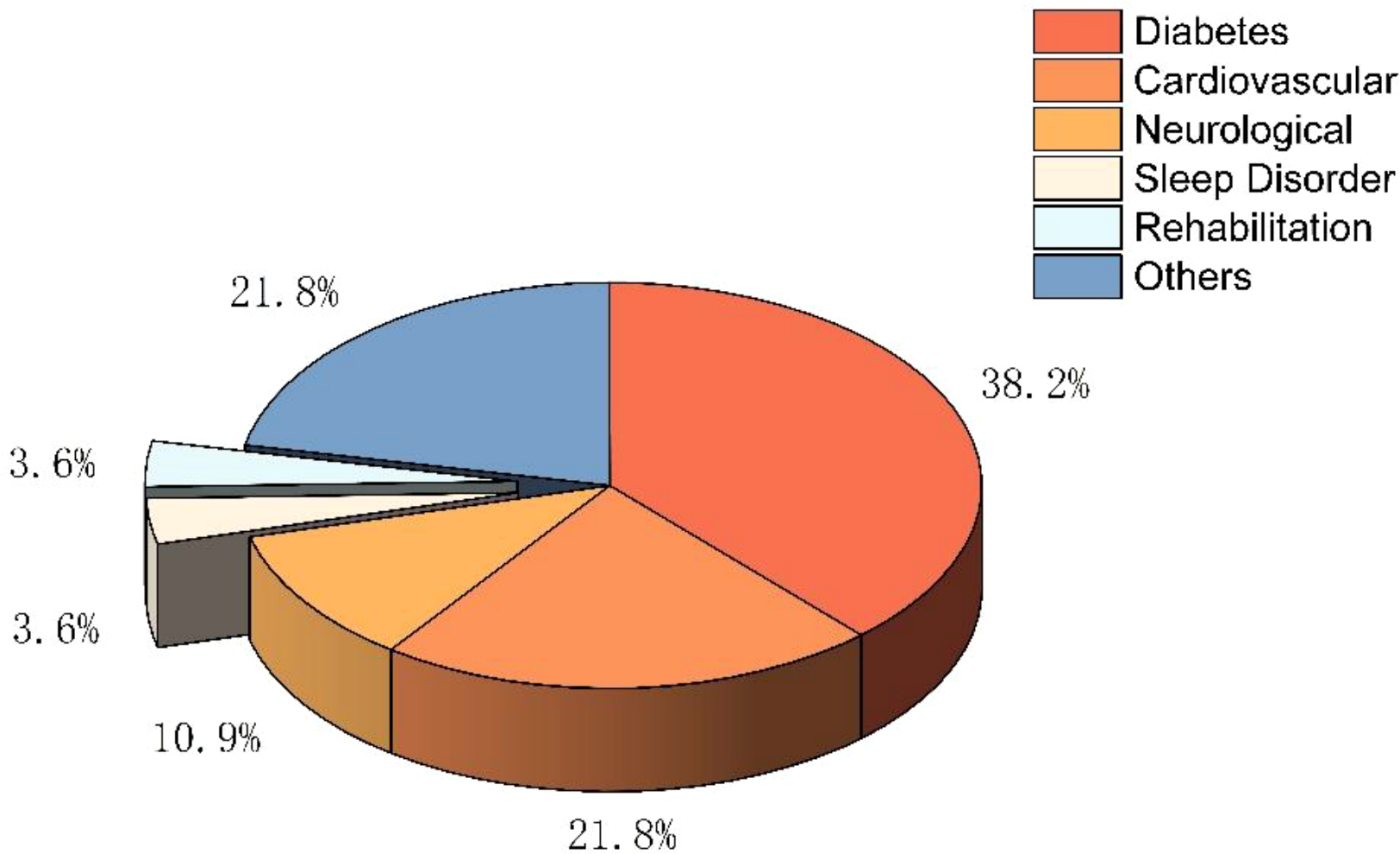
Clinical domains represented in the studies included in this review.

This 3-dimensional pie chart (**Figure 3**) illustrates the distribution of clinical application areas across the studies employing entropy-based heart rate variability (HRV) analysis. Diabetes-related research constitutes the largest proportion at 38.2%, followed by cardiovascular applications (21.8%) and neurological conditions (21.8%). Studies addressing sleep disorders and rehabilitation each account for 3.6% of the total, while the remaining 10.9% fall into other miscellaneous clinical domains. This distribution demonstrates the predominant focus on metabolic and cardiovascular dysfunction in existing entropy-based HRV research and highlights emerging applications beyond these traditional areas.

**Figure 4:**
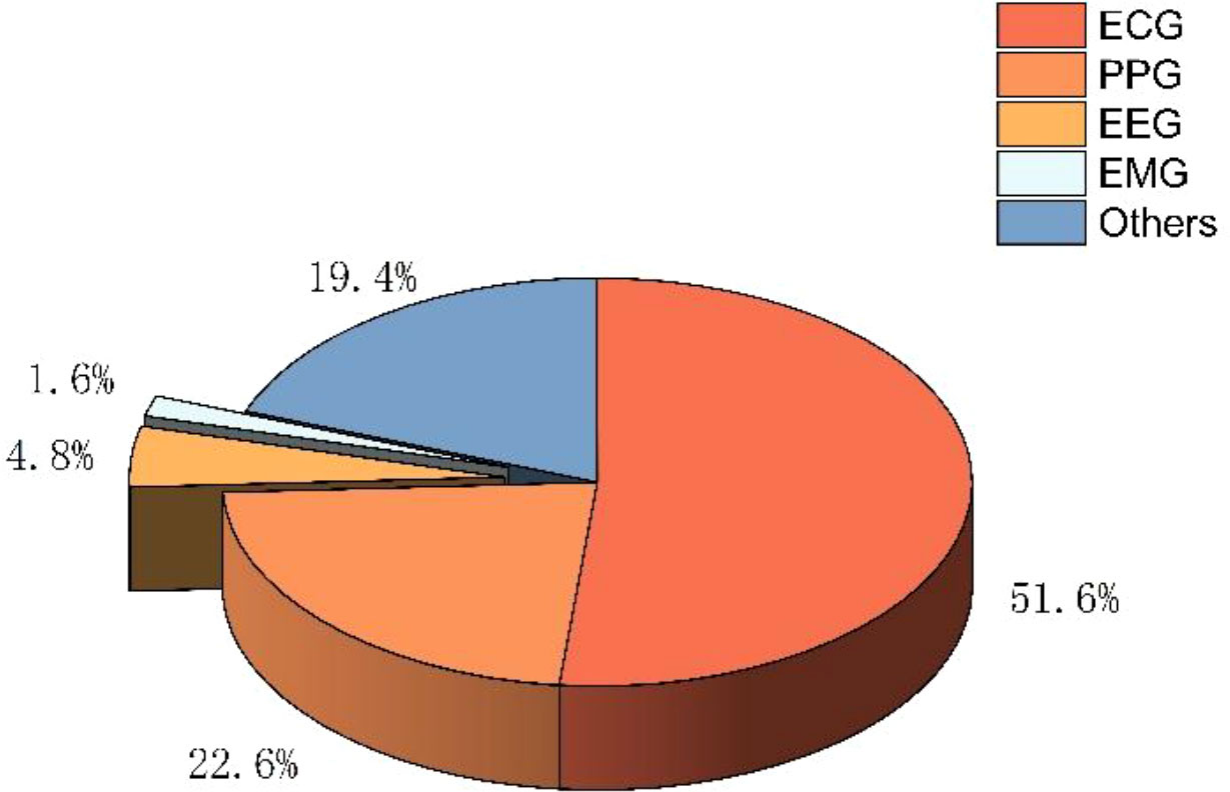
Distribution of physiological signal sources used for entropy-based analysis in the reviewed studies.

This 3-dimensional pie chart (**Figure 4**) summarises the types of physiological signals employed across the included studies. Electrocardiography (ECG) is the predominant data source, representing 51.6% of all applications. Photoplethysmography (PPG) accounts for 22.6%, followed by electroencephalography (EEG) at 4.8% and electromyography (EMG) at 1.6%. The remaining 19.4% corresponds to other physiological signals, including multimodal and less commonly used biosignals. This distribution underscores the central role of ECG in entropy-based heart rate variability assessment while reflecting growing interest in alternative and complementary signal modalities.

Across the studies reviewed, notable trends emerge regarding the selection of entropy methods, clinical application areas, and the physiological signal sources used for analysis. First, the distribution of entropy techniques demonstrates substantial methodological diversity. While multi-scale entropy (MSE) accounts for a significant portion of applications, a large proportion of studies employ other established and emerging entropy measures, including approximate entropy, fuzzy entropy, permutation entropy, and novel indices such as percussion entropy. This heterogeneity highlights both the maturity of traditional complexity metrics and the increasing exploration of advanced non-linear approaches designed to capture subtle autonomic alterations in aging and diabetic populations.

Second, the clinical domains represented in the literature reveal a predominant focus on diabetes, followed by cardiovascular and neurological conditions. These findings reflect the central role of autonomic dysfunction in the pathophysiology of these disorders and underscore the clinical relevance of entropy-based HRV analysis in identifying early autonomic impairment. Smaller but meaningful contributions arise from studies involving sleep disorders, rehabilitation contexts, and other miscellaneous conditions, indicating expanding interest in broader diagnostic and rehabilitation applications.

Third, the review shows that ECG remains the principal physiological signal source for entropy computation, underscoring its long-standing role in autonomic assessment. However, the increasing adoption of alternative biosignals such as photoplethysmography (PPG), EEG, EMG, and multimodal data reflects a growing movement toward flexible and application-specific monitoring strategies. This trend aligns with advancements in wearable technology and sensor integration, enabling broader accessibility to HRV-based autonomic assessment in both clinical and real-world environments.

Collectively, these findings demonstrate a rapidly evolving research landscape characterised by methodological innovation, expanding clinical utilisation, and diversification of physiological data sources. Such developments reinforce the potential of entropy analysis as a robust and versatile approach for assessing autonomic dysfunction, particularly in aging and diabetic populations.

## 4 Discussion

Entropy-based heart rate variability (HRV) analysis is a promising approach for understanding autonomic dysfunction, particularly in aging and diabetic populations. This review evaluates its applications, unresolved questions, and future directions, highlighting how advancements in computational techniques and novel indices are transforming HRV assessment.

### 4.1 Advances in entropy analysis for autonomic dysfunction

The complexity of autonomic dysfunction in aged and diabetic populations necessitates sensitive and robust assessment tools. Traditional HRV measures, including time-domain and frequency-domain methods, provide valuable information on autonomic regulation but may not fully capture the nonlinear and multi-scale dynamics of cardiovascular control. In this context, entropy-based metrics have been proposed as complementary approaches that can offer additional insights into the complexity of physiological regulation. Entropy analysis overcomes these limitations by quantifying the unpredictability and complexity of heart rate signals, providing a more comprehensive understanding of autonomic health (Castiglioni et al., 2022).

Emerging indices, including multi-scale entropy (MSE), permutation entropy (PermEn), and baroreflex entropy index (BEI), have significantly improved diagnostic capabilities. For example, MSE assesses HRV across multiple time scales, detecting both short-term fluctuations and long-term trends (Valencia et al., 2009; Ho et al., 2011; Frassineti et al., 2021). PermEn, by focusing on ordinal patterns rather than amplitude, demonstrates resilience to noise and non-stationarity, making it particularly suitable for diabetic populations in which ECG signal quality may vary (Bian et al., 2012). BEI offers insights into baroreflex sensitivity, a key marker of autonomic regulation (Yang et al., 2024). Despite these advancements, several methodological gaps persist. Variability in entropy calculation methods, data preprocessing, and parameter selection limits comparability across studies (Cornforth et al., 2014). To address this issue, future research should prioritise the standardisation of entropy metrics, ensuring consistency in clinical and research settings.

### 4.2 Unresolved questions in entropy metrics

While entropy metrics have shown potential in short-term studies, their long-term prognostic value remains underexplored. For example, MSE has demonstrated utility in predicting outcomes in patients with heart failure (Costa et al., 2002; Costa et al., 2005; Ho et al., 2011), but its applicability to long-term diabetes complications, such as diabetic autonomic neuropathy (DAN), is unclear. Longitudinal studies are essential to establish entropy metrics as reliable indicators of chronic autonomic dysfunction. Additionally, the interplay of comorbidities such as hypertension, cardiovascular disease, and metabolic syndrome complicates the interpretation of entropy metrics. Aging and diabetes are often accompanied by overlapping pathophysiological changes that influence HRV (Mehanna and Jankovic, 2024). Advanced analytical techniques, such as machine learning, can help isolate diabetes-specific effects on entropy metrics, enhancing their diagnostic specificity.

The physiological mechanisms underlying entropy changes also warrant further investigation. Inflammatory processes, commonly associated with diabetes and aging, significantly affect autonomic function (Vinik et al., 2013). Studies have linked inflammation to reduced baroreflex sensitivity and altered HRV. Exploring how entropy metrics reflect these changes could improve our understanding of autonomic regulation in pathological states (Suarez-Roca et al., 2024).

### 4.3 Impact of confounding factors on HRV analysis

The clinical utility of entropy metrics is often challenged by confounding factors, including medications, metabolic variability, and signal acquisition methods. For instance, β-blockers, commonly used in cardiovascular disease management, may affect HRV (Niemelä et al., 1994). However, some entropy measures, such as specific MSE scales, retain prognostic value independent of medication effects (Rueda-Ochoa et al., 2024). Signal acquisition variability, such as differences between electrocardiography (ECG) and photoplethysmography (PPG), further complicates entropy analysis (Esgalhado et al., 2022). While PPG offers a non-invasive alternative for continuous monitoring, its susceptibility to motion artifacts and lower signal fidelity poses challenges (Lee et al., 2020). Recent advancements in signal processing, including adaptive filtering and data fusion, have improved the reliability of PPG-derived HRV metrics, making them viable for broader applications (Jong et al., 2017).

To address these challenges, future studies should implement standardised protocols for signal acquisition and pre-processing. Incorporating advanced statistical methods, such as predictive regression models, can further mitigate the impact of confounders and improve the reliability of entropy metrics.

### 4.4 Computational techniques transforming HRV assessment

The integration of advanced computational techniques has revolutionised HRV analysis, enabling the extraction of deeper insights from entropy metrics. Machine learning algorithms, such as convolutional (CNNs) and recurrent neural network (RNNs), can identify complex patterns in HRV data, improving diagnostic accuracy (Matuz et al., 2022). These models have been particularly effective in distinguishing between individuals with and those without diabetes, highlighting their utility in large-scale screening (Rustam et al., 2024). Data fusion approaches, combining multiple physiological signals from ECG, PPG, and pulse wave velocity (PWV), provide a holistic view of autonomic function (Pilz et al., 2024). For instance, integrating PWV with entropy analysis enhances the assessment of arterial stiffness, a critical factor in cardiovascular risk stratification (Starzak et al., 2022). Similarly, crest time analysis adds valuable information about arterial compliance, complementing traditional HRV metrics (Kyriacou and Allen, 2021). Real-time signal processing platforms and wearable devices further expand the potential applications of entropy-based HRV analysis. These technologies enable continuous monitoring, providing immediate feedback to patients and clinicians. Such capabilities are particularly beneficial for managing chronic conditions, such as diabetes and hypertension, in which real-time insights can guide personalised interventions.

### 4.5 Clinical applications and training needs

Wearable devices equipped with entropy-based HRV monitoring capabilities have opened new avenues for personalised healthcare. These devices allow for continuous, non-invasive assessment of autonomic function, facilitating early detection of complications such as DAN (Li et al., 2023). The integration of wearable technology with telemedicine platforms further enhances care quality, enabling remote monitoring and timely interventions (Zhao et al., 2024). However, the adoption of entropy analysis in clinical practice requires specialised training for healthcare professionals. The mathematical complexity of entropy metrics and their interpretation in the context of autonomic dysfunction demand a solid understanding of both theory and application. Structured training programs and certification processes could bridge this gap, ensuring clinicians are equipped to use these advanced tools effectively (Klemann et al., 2024). To maximise clinical utility, future research should focus on developing user-friendly software and platforms that simplify entropy analysis. Collaborative efforts between engineers, clinicians, and researchers are essential to ensure that these tools meet clinical needs without compromising accuracy.

### 4.6 Future directions

Looking ahead, entropy analysis holds significant promise for advancing the understanding and management of autonomic dysfunction. Longitudinal studies are critical for establishing the prognostic value of entropy metrics in chronic conditions. Integrating novel computational methods, such as artificial intelligence and real-time data processing, will further enhance the precision and applicability of HRV analysis. Innovative technologies, such as wearable ECG devices capable of continuous entropy monitoring, represent a key area of development. These devices could enable home-based care, improving accessibility and reducing the burden on healthcare systems. By facilitating early detection and personalised management, wearable technology has the potential to transform the landscape of autonomic dysfunction assessment (Alugubelli et al., 2022). Additionally, exploring the physiological underpinnings of entropy changes will provide valuable insights into the mechanisms of autonomic regulation. Such research could inform the development of targeted interventions, improving outcomes for individuals with diabetes and age-related autonomic decline.

In summary, entropy analysis represents a pivotal advancement in HRV assessment, offering a nuanced understanding of autonomic function in aging and diabetic populations. By addressing existing gaps in methodology, standardisation, and training, this field can achieve its full potential in clinical practice. Future research should focus on refining entropy metrics, developing accessible technologies, and fostering interdisciplinary collaboration to improve diagnostic accuracy and patient outcomes.

## 5 Conclusions and future work

In this systematic review, we highlight the significant potential of entropy analysis for assessing HRV in older and diabetic populations. By capturing the complexity and irregularity of heart rate signals, entropy analysis offers a nuanced understanding of autonomic function that traditional time-domain and frequency-domain methods may overlook. Our exploration into the application of entropy analysis has revealed several key insights and unresolved questions, emphasising the need for further research and refinement in this field. First, the long-term prognostic value of ECG entropy analysis in older and diabetic populations remains insufficiently explored. Although existing studies provide valuable insights into short-term indicators and acute changes, long-term follow-up data are lacking. Future research should prioritise longitudinal studies to establish the prognostic implications of entropy metrics over extended periods, thereby enhancing our understanding of their utility in predicting cardiovascular outcomes and guiding clinical decision-making. Second, the impact of multiple comorbidities, such as hypertension and cardiovascular diseases, on ECG entropy metrics needs to be thoroughly assessed. Older individuals and those with diabetes often present with complex clinical profiles, and accounting for these confounding factors is crucial to obtain accurate and meaningful results. Future studies should employ robust methodologies to isolate the effects of diabetes on HRV entropy and explore the interplay between various comorbid conditions and autonomic dysfunction. Third, the underlying mechanisms through which changes in entropy reflect autonomic dysfunction are incompletely understood. Investigating the physiological and pathophysiological processes that drive alterations in entropy metrics will provide deeper insights into the autonomic regulation of the heart. Integrating advanced computational techniques, such as machine learning and artificial intelligence, may offer novel approaches to deciphering these complex mechanisms and identifying predictive patterns in HRV data. Moreover, the level of training and expertise required for healthcare professionals to accurately interpret and utilise entropy analysis is an often-overlooked aspect. Ensuring adequate training for clinicians and researchers in the application of entropy methods is imperative for the reliable and consistent use of this analytical tool. Developing standardised training programs and certification processes could enhance the proficiency of healthcare providers and facilitate the broader adoption of entropy analysis in clinical practice. In the future, studies should also focus on developing innovative methods to enhance the quality of diabetes care. The integration of wearable ECG devices capable of real-time entropy analysis promises home-based autonomic function assessment. Such advancements could enable continuous monitoring of HRV and provide early detection of autonomic dysfunction, thereby facilitating timely interventions and personalised management strategies.

Conclusively, entropy analysis represents a promising avenue for assessing autonomic function in older and diabetic populations. Herein, we underscore the need for further research to address the existing lacunae and enhance the clinical utility of entropy metrics. By advancing our understanding of autonomic dysfunction and its implications for cardiovascular health, we can improve diagnostic accuracy, advise on therapeutic approaches, and ultimately contribute to better health outcomes for individuals with diabetes and age-related autonomic decline. Future research endeavours should aim to explore innovative technologies and methodologies, fostering a comprehensive and integrative approach to autonomic function assessment.
